# Supplementary material for: Structural Variant Disrupting the Expression of the Remote FOXC1 Gene in a Patient with Syndromic Complex Microphthalmia
Source: Int J Mol Sci. 2024 Feb 25;25(5):2669. doi: 10.3390/ijms25052669 (PMC10931988; doi:10.3390/ijms25052669)
Supplement: Supplementary file 1 [file ijms-25-02669-s001.zip › ijms-2853855-supplementary.pdf]

International Journal of *Molecular Sciences*

MDPI

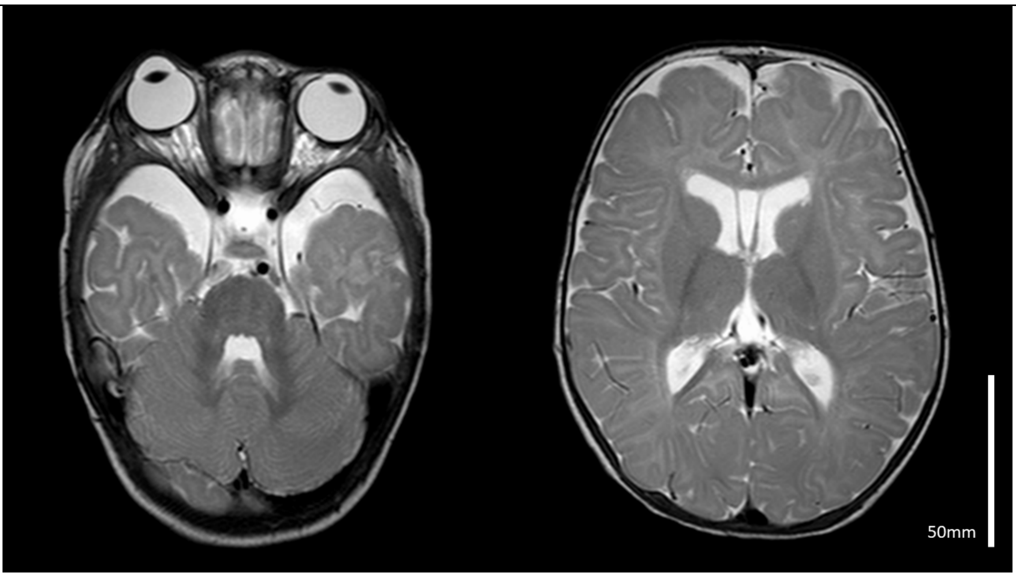

Figure S1: Cerebral MRI (T2 weighted) performed at 6-mounth-old under general anesthesia showing right microphthalmia, presence of optic nerves (left) and cysts of the septum pellucidum (right).

A. Results Overview head_neck

Gene	NeuralCrestEarly	NeuralCrestLate	PalateCS20	Pathomechanism
FOXC1	LOF: 0.81	LOF: 1	LOF: 1	Long-Range
GMDS	LOF: 0.83	LOF: 0.83	LOF: 0.83	Gene Truncation
CDYL	-	-	-	Gene Truncation
RPP40	-	-	-	Long-Range

B.

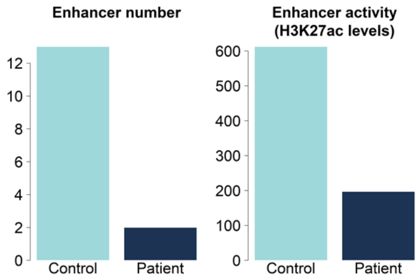

Figure S2: Prediction of the impact of the 6p25.1p25.3 inversion using POSTRE software. **A**. Results Overview for head and neck. **B**. Predicted impact on FOXC1 enhancer number and activity.
